# The Gonococcus of Neiser

**Published:** 1887-06

**Authors:** C. W. Allen


THE GONOCOCCUS OF NEISER.

It has become fairly well established that there is a micro-organism peculiar to the gonorrhœa, and that it can be recognized with certainty in all cases. This micro-organism is the genococcus of Neiser (*Centralblatt fur Deutsche Mediziniche Wissenschaft, No. 28, 1879*), since thoroughly confirmed by Bokai, Bochkhart, Sternberg, Keyser, Zeissl, and many others less well-known; and the process for its unmistakable detection is that of Dr. Gabriel Roux, of Paris. This process is based upon the fact that, after treatment of a slide prepared according to the steps of Gram's method, all gonocci are entirely decolorized by the washing in alcohol which follows the treatment with the iodo-iodide of potassium solution. The process in full is as follows: A drop of pus is spread into a thin layer between two glass slides and allowed to dry in the air. A drop of a solution of methyl-blue in aniline water is placed upon it for a moment and washed off with a stream from a wash bottle, and a few drops of iodo-iodide solution is placed upon and allowed to remain for a few minutes. This fixes the color on micro-organisms in general. Gram's solution is then washed off with water, and while it is still wet a cover glass is placed upon it and it is examined with an oil immersion lens. If micro-organisms resembling gonococci are found, the slide is decolorized as thoroughly as possible with absolute alcohol, and reexamined in water, when all gonococci will be found to have disappeared whilst the other forms of micro-organisms still remain. If desired the pus cells may be brought out again by applying a solution of eosin. As a result of a very large number of examinations of pus, simple and gonorrhœal, it may be said that there is no gonorrhœa without gonococci. The practical value of this test is very great. It will occasionally clear up the diagnosis in cases of vaginal secretions; it may declare the origin of a case of ophthalmia neonatorum; it may have medico-legal value in a case of rape, it may favorably determine the nature of a vaginitus in little girls, and it will declare the nature of a urethral discharge in a male.

It is still undecided whether the gonococcus is the materies morbi of gonorrhœa, but the last successful inoculation, by Bumm appears to have been done with all necessary precautions. In the present state of our knowledge the following conclusions are justifiable.

1. A micrococcus is found in gonorrhœa, and gonorrhœal diseases which is not found elsewhere.

2. It is in all probability the specific microbe of gonorrhœa.

3. Its discovery has been of great practical value, especially as regards diagnosis and prophylaxis.

4. The method proposed by Roux furnishes the most convenient means of proving the identity of the gonococcus in doubtful cases.

5. As regards treatment the discovery cannot as yet be said to have produced any decided advances.—*C. W. Allen, M. D., in Journal of Cutaneous and Genito-Urinary Diseases, March, 1887.*

